# The clinical relevance of axillary reverse mapping (ARM): study protocol for a randomized controlled trial

**DOI:** 10.1186/1745-6215-14-111

**Published:** 2013-04-25

**Authors:** Elisabeth G Klompenhouwer, Paul D Gobardhan, Martinus A Beek, Adri C Voogd, Ernest JT Luiten

**Affiliations:** 1 Department of Radiology, Catharina Hospital Eindhoven, Eindhoven, the Netherlands; 2 Department of Surgery, Amphia Hospital, Molengracht 21, 4818, CK Breda, The Netherlands; 3 Department of Epidemiology, Maastricht University, Maastricht, The Netherlands; 4 Faculty of Health Medicine and Life Sciences, Maastricht University, Maastricht, The Netherlands; 5 Research Institute Growth and Development (GROW), Maastricht University, Maastricht, The Netherlands; 6 Eindhoven Cancer Registry, Comprehensive Cancer Center South, Eindhoven, The Netherlands

**Keywords:** Breast cancer, Axillary lymph node dissection, Breast cancer-related lymphedema, Axillary reverse mapping

## Abstract

**Background:**

Axillary lymph node dissection (ALND) in patients with breast cancer has the potential to induce side-effects, including upper-limb lymphedema. Axillary reverse mapping (ARM) is a technique that enables discrimination of the lymphatic drainage of the breast from that of the upper limb in the axillary lymph node (LN) basin. If lymphedema is caused by removing these lymphatics and nodes in the upper limb, the possibility of identifying these lymphatics would enable surgeons to preserve them. The aim of this study is to determine the clinical relevance of selective axillary LN and lymphatic preservation by means of ARM. To minimize the risk of overlooking tumor-positive ARM nodes and the associated risk of undertreatment, we will only include patients with a tumor-positive sentinel lymph node (SLN). Patients who are candidates for ALND because of a proven positive axillary LN at clinical examination can be included in a registration study.

**Methods/design:**

The study will enroll 280 patients diagnosed with SLN biopsy-proven metastasis of invasive breast cancer with an indication for a completion ALND. Patients will be randomized to undergo standard ALND or an ALND in which the ARM nodes and their corresponding lymphatics will be left *in situ*. Primary outcome is the presence of axillary surgery-related lymphedema at 6, 12, and 24 months post-operatively, measured by the water-displacement method. Secondary outcome measures include pain, paresthesia, numbness, and loss of shoulder mobility, quality of life, and axillary recurrence risk.

**Discussion:**

The benefit of ALND in patients with a positive SLN is a subject of debate. For many patients, an ALND will remain the treatment of choice. This multicenter randomized trial will provide evidence of whether or not axillary LN preservation by means of ARM decreases the side-effects of an ALND. Enrolment of patients will start in April 2013 in five breast-cancer centers in the Netherlands, and is expected to conclude by April 2016.

**Trial registration:**

TC3698

## Background

Axillary lymph node dissection (ALND) for patients with breast cancer has the potential to cause side-effects, including pain, numbness or paresthesia, arm/shoulder mobility restriction, and lymphedema [1]. Arm lymphedema has been documented in 7 to 77% of patients who undergo ALND [2,3]. The incidence of lymphedema depends on whether ALND has been combined with subsequent radiotherapy, but also on the definition of ALND used.

Sentinel lymph node biopsy (SLNB) represents the standard of care for axillary node staging in patients with early-stage, clinically node-negative breast cancer (cT1 to 2N0). The goals of SLNB are to reduce the morbidity from breast-cancer surgery by avoiding unnecessary ALND and to improve staging of the regional LNs. However, in a selected group of patients, ALND is still indicated.

Axillary reverse mapping (ARM) is a recently developed technique that enables surgeons to discriminate the lymphatic drainage pattern of the breast from that of the upper limb. The concept of ARM is to map the drainage of the upper limb to determine the anatomical variation in these lymphatics and thus create a road map for their preservation. If lymphedema is caused by removing the lymphatics and nodes of the upper limb, the possibility of identifying these lymphatics would enable surgeons to preserve them [4]. The ARM technique can be performed by using blue dye, fluorescent dye, or a radioisotope. In the past few years, several groups have reported their first experiences with this relatively new technique [5-13]. A review by Ngochi on this topic [14] clearly described the ARM techniques that are currently available. Studies on ARM using blue dye reported identification rates varying between 50 and 89% [5-10], and for isotope and fluorescent dyes, these rates are 91 to 100% [11,13] and 88% [12] respectively. The latter two visualization techniques have the advantage of not leaving a ‘blue tattoo’ on the patient’s skin. However, there is limited information on the use of isotopes and fluorescent dyes, and they require the use of expensive equipment during surgery.

Between October 2009 and June 2011, we performed a pilot study using blue dye for visualizing LN drainage of the upper limb [15]. Patients with invasive breast cancer and an indication for ALND were enrolled in the study, and these comprised 50 patients with a tumor-positive SLN and 43 patients who had axillary LN metastases proven by pre-operative cytology. During surgery, ARM nodes were identified and removed first, followed by ALND (at least level I to II). No significant differences were seen in the visualization rate of ARM nodes between the groups (86 and 94% respectively, *P* = 0.196). In the group of patients with a positive SLN, none of the ARM nodes contained metastases, whereas 11 (22%) of the ARM nodes in the group with axillary metastases proven by pre-operative cytology contained metastases (*P* = 0.001). Patients receiving neoadjuvant chemotherapy had a significantly lower risk of additional axillary LN metastases (24.6 versus 44.4%, *P* = 0.046). These results are largely in accordance with other studies. Boneti *et al*. [8], Thompson *et al*. [7], and Casbona *et al*. [10] found no tumor deposits in the ARM nodes even when the patients had positive axillary nodes in the initial series. However, Nos *et al*. [6] found metastases in 14% of the ARM nodes (3 of 21 patients), all of which were associated with extensive axillary LN metastasis. Noguchi *et al*. [12] found ARM node metastases in three of seven patients who underwent ALND, and all three patients had a clinically positive axilla (N1/N2). Based on our results and those from the literature, we conclude that the ARM procedure using blue dye is technically feasible and has a high visualization rate, and that its use might be considered in patients with a positive SLN. Nevertheless, more research is needed to determine the safety of ARM in patients with clinically positive LNs.

Despite these promising results, there are some problems that need to be considered before ARM can be used in routine clinical practice. The currently available techniques are insufficient to identity the upper-limb LNs in some patients. The relevance of the ARM procedure is based on the assumption that the lymphatic pathways from the upper limb are not involved in metastasis of the primary breast cancer [5]; however, in a minority of patients, the SLN draining of the breast may be the same as the ARM node draining the upper limb. This may explain why the ARM nodes may be involved with metastatic foci in patients with clinically axillary LN metastases. These issues may represent an important drawback for the implementation of the ARM procedure.

Boneti *et al*. recently published a phase II trial of ARM with promising results [16]. The study showed that preserving the ARM nodes in a clinically negative axilla is safe, and results in a low incidence of post-operative lymphedema in patients undergoing ALND and SLNB. However, further studies are needed before this technique can be adopted as a standard procedure during complementary ALND (cALND) in the surgical treatment of breast cancer.

In this paper, we present the design for a multicenter randomized controlled trial to determine the clinical relevance and safety of selectively sparing upper-limb axillary LNs and their corresponding lymphatics by means of ARM. To minimize the risk of under-treatment due to non-removal of possible ARM node metastases, we will only include patients based on a tumor-positive SLN.

## Methods/design

The aim of the ARM trial is to determine the usefulness of the ARM technique in identifying and sparing the upper limb-related axillary LNs, and its ability to reduce the risk of lymphedema of the upper limb. After determining the indication for a cALND basis on a tumor-positive SLN, patients will be randomly allocated to one of two groups: ALND with preservation of the upper limb-related axillary LNs (ARM-ALND) and a level I-II ALND (standard ALND).

**Figure 1:**
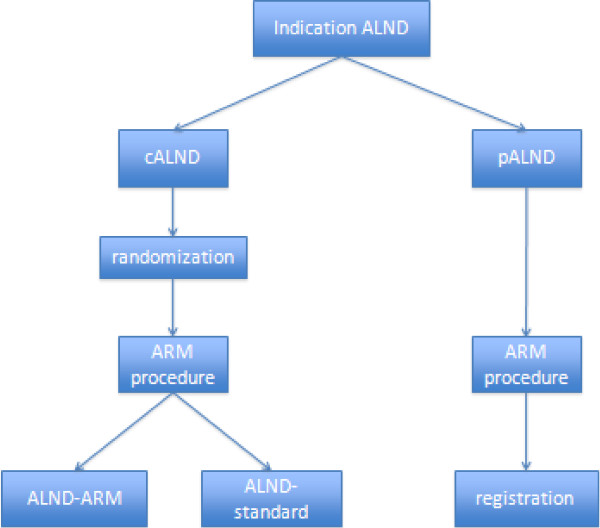
**Flow chart of axillary reverse mapping (ARM) trial. **ALND, Axillary lymph node dissection; cALND, complementary axillary lymph node dissection based on a tumor-positive sentinel node; pALND, primary axillary lymph node dissection based on a tumor-positive axilla proven by pre-operative cytology; ARM-ALND axillary lymph node dissection with preservation of the upper limb-related axillary lymph nodes; standard ALND, level I-II axillary lymph node dissection.

Patients who are candidates for a primary axillary lymph node dissection (pALND), based on axillary metastases proven by pre-operative cytology, can be included in the registration study to confirm the feasibility of the procedure and to perform a further subgroup analysis. A schematic overview of the trial is given in Figure 1.

### Participants

Female patients aged over 18 years presenting with invasive breast cancer and an indication for a cALND based on a positive SLN will be deemed eligible for the trial. The indication for the ALND should be made by a multidisciplinary team including an oncologic surgeon, a medical oncologist, a pathologist, a radiologist, and a radiotherapist. Informed consent will be obtained from all patients. Exclusion criteria are: indication for pALND based on a clinical positive axilla; contraindication for SLNB; an adverse event during the previous SLNB; history of breast cancer or axillary surgery; or pregnancy.

Patients who undergo an ALND based on a clinically positive axilla can participate in the registration study, in which patients will undergo ARM during ALND. In this group of patients, no attempt will be made to spare LNs or lymphatics.

### Objectives

The hypothesis is that patients undergoing ARM-ALND will have fewer post-operative complications than patients undergoing standard ALND. The post-operative complications that will be measured are the occurrence of breast cancer-related lymphedema (BCRL) and paresthesia/numbness, pain, and shoulder immobility. The hypothesis is that a reduction in the risk of post-operative complications in patients undergoing ARM-ALND will result in a better quality of life compared with those undergoing standard ALND. Based on the results of our feasibility study, in which no LN metastasis where found in the patients with tumor-positive SLN, it is expected that the axillary recurrence risk in the ARM-ALND group will be comparable with the risk in the standard ALND group.

#### Research question

1. Will there be fewer post-operative complications after ARM-ALND compared with standard ALND in patients with an indication for a cALND based on a positive SLNB? Post-operative complications are expected to include the following. primary complication: BCRL; secondary: pain, paresthesia/numbness, and loss of shoulder mobility.

2. Will the possible reductions in these post-operative complications have a positive effect on the quality of life (QoL)?

3. Will patients undergoing ARM-ALND have a similar risk of axillary recurrence as the patients undergoing standard ALND?

### Randomization

Randomization will be performed using a web-based randomization system, and will be stratified by center. This randomization application and the internet-based help service will be available 24 hours a day, 7 days a week. Because of practical considerations, the randomization will take place as soon as the patient has given written informed consent. Based on our previous experience with the ARM procedure, we expect that identification or preservation of the ARM nodes/lymphatics will not be possible in 5 to 15% of the randomized patients. In such cases, the standard ALND will be performed, if possible, and the ARM nodes/lymphatics will be separately removed and sent for analysis. These patients will be included in the analysis in accordance with the intention-to-treat principle, but excluded from the sensitivity analysis. (Figure 1) (more information can be found at http://www.armstudie.nl).

### Interventions

Surgeons familiar with the ARM procedure will perform the ALND. In our feasibility study (Amphia Hospital Breda, the Netherlands), the learning curve for performing the ARM procedure was very small (barely measureable), and the overall identification rate of the upper-limb LNs was up to 94%. Based on these results, we consider an oncologic breast surgeon should be capable of performing an ARM procedure once they have performed five procedures under the supervision of one of the oncologic surgeons (EL or PG) from the Amphia Hospital Breda.

The procedure will be performed under general anesthesia. For the procedure, approximately 2 ml of blue patent V dye (Laboratoire Guerbet, Aulnay-sous-Bois, France) from a stock solution of 25 mg/ml is injected subcutaneously into the inner arm, along the medial intermuscular groove of the ipsilateral arm. This dye is identical to the dye used to perform an SLNB. After injection, the site of injection is massaged and the arm elevated for a few minutes to enhance lymphatic drainage of this limb. Next, the patient is covered with sterile drapes, and the ALND can be started.

**Figure 2:**
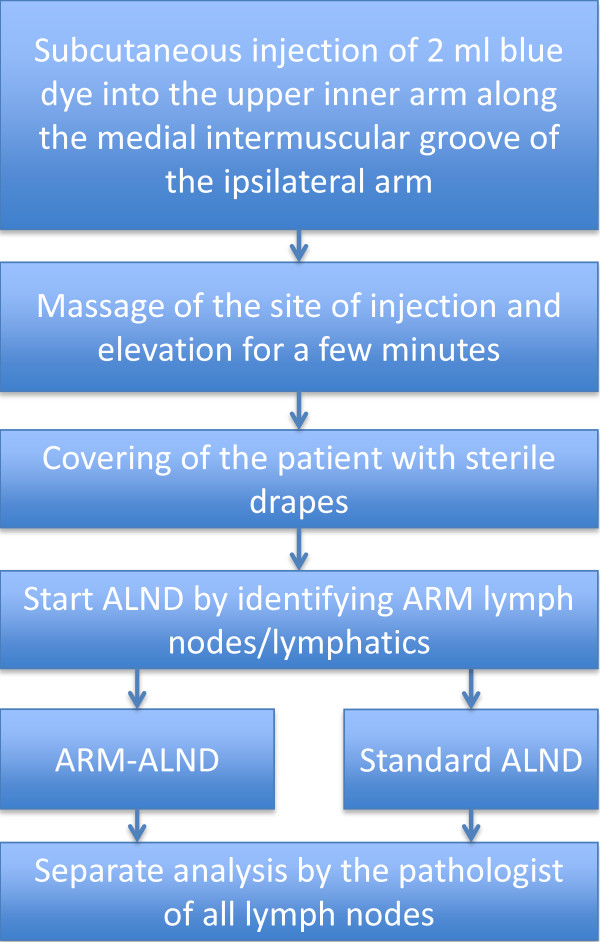
Axillary reverse mapping (ARM) procedure.

The technical part of the operation is identical to a normal level I to II ALND, except for the first part of the operation. After incision, the blue-stained LNs and lymphatics are identified. In the ARM-ALND group, the blue-stained LNs and lymphatics will be spared, then a normal ALND will be performed. In the standard ALND group, a normal ALND will be performed without sparing the upper-limb lymphatics and LNs. The pathologist will separately analyze the LNs (level I to II nodes and ARM nodes, Figure 2).

### Settings and location

The study is a multicenter randomized controlled trial, which will be conducted in the Netherlands. Already five dedicated breast-cancer centers in the Netherlands are willing to participate. These are: Amphia Hospital, Breda; Catharina Hospital, Eindhoven; Haga Hospital, The Hague; Hospital Gelderse Vallei, Ede; and Albert Schweitzer Hospital, Dordrecht.

Patients eligible for inclusion will be enrolled by the breast surgeon at the outpatient clinic, after providing informed consent.

### Primary and secondary outcome measures

The primary outcome will be the occurrence of lymphedema in the upper limb. Lymphedema will be measured s at 6, 12 and 24 months, using the following two techniques.

1) The water-displacement method. This method is regarded as the gold standard for the measurement of lymphedema. For the assessment of upper-limb volume, an arm volume meter will be used (NV Varitex Haarlem, The Netherlands). This method has high reproducibility [17]. Both upper limbs will be measured pre-operatively and post-operatively. Lymphedema is defined as a difference in limb volume of greater than 200 ml between the treated and untreated side, as described by Lopez Penha *et al*. [18].

2) Circumference method. The arm circumference will be measured at four sites: metacarpophalangeal joints, wrist, 100 mm distal from the lateral epicondyle, and 150 mm proximal of the epicondyle. A difference of more than 20 mm between the affected and contralateral arm is considered clinically significant.

The secondary endpoints will be measured at 6, 12, and 24 months and comprise the following:

1) Paresthesia/numbness. This will be assessed with the aid of a standardized questionnaire developed for patients with breast cancer [19].

2) Pain. This will be measured using a visual analog scale.

3) Loss of shoulder mobility. The shoulder function of the affected upper limb will be compared with the unaffected one. Loss of abduction of more than 20 degrees will be considered abnormal.

4) Quality of life. This will be assessed by a standardized questionnaire developed for patients with breast cancer [21]. This large questionnaire includes the WHOQOL-Bref.

5) Locoregional recurrence rate: This will be assessed by a physical examination during follow-up. In cases where there is suspicion of recurrence, ultrasonography (with or without biopsy) will be performed.

### Data collection

A nurse practitioner or clinical investigator (depending on the local organization of the participating hospital) will be responsible for the pre-operative and post-operative data collection. The surgeon who performed the ALND will be responsible for the peri-operative data collection. Data will be stored anonymously under a unique trial number. The principal investigators (EL and PG) will have access to the data. All data will be collected in an online case report form and an online participant questionnaire (PQ). The timetable and source of the data collection is listed in Table 1.

**Table 1:** Data collection

**Data**	**Source**	**Pre-operative**	**Peri-operative**	**Follow-up at 6, 12, and 24 months**
Baseline characteristics	CRF	x		
Medical history	CRF	x		
Pre-operative tumor status/markers	CRF	x		
Operation data including:	CRF		x	
Registration				
Randomization				
Post-operative complications	CRF/PQ	x^a^		x
Quality of life	PQ			x
Axillary recurrence rate	CRF			x

CRF, case report form; PQ, participant questionnaire.

^a^The volume, circumference and mobility of the arm will be measured pre-operatively.

### Blinding

The qualified oncologic breast surgeon will perform the procedure and therefore cannot be blinded. The patient and the person responsible for the post-operative data collection and measurements will not be aware of the performed procedure (double-blind procedure).

### Statistics

#### Sample size

The clinically relevant amount of lymphedema is stated as a volume difference of 200 ml or more between both arms. Primary research shows that the risk of BCRL is 15% in patients undergoing an ALND. It is expected that ARM-ALND will reduce the risk to 5%. Based on these assumptions, we will need to study 140 subjects in the standard ALND group and 140 subjects in the ALND-ARM group to be able to reject the null hypothesis that the failure rates for experimental and control subjects are equal with a probability (power) of 0.8. The Type I error probability associated with this test of the null hypothesis is 0.05.

#### Data analysis

We will use an uncorrected χ^2^ statistic to test the null hypothesis for the proportion of patients developing lymphedema in the upper limb. These tests will be performed after 6, 12, and 24 months. We are interested in the course of the development of lymphedema, based on the measurements at each of the three specified time points. As development of lymphedema has been reported to occur 2 or more years after surgery, the measurement of lymphedema at 24 months is considered our primary endpoint.

To compare continuous data, such as pain and quality of life, analysis of covariance (ANCOVA) will be performed, using baseline values as a covariate. Separate analyses will be performed for the three time points (6, 12 and 24 months). The local recurrence rate in both groups will be analyzed by the Kaplan-Meier method and compared with the log-rank test. A repeated-measures analysis will be performed to compare the changes in continuous data between the two groups, until 2 years after treatment. It is expected that not only will the prevalence of lymphedema be higher in the standard ALND arm, but also that it will occur earlier and will be more severe. To test this hypothesis, interaction terms between treatment and time will be included in the repeated-measures analysis.

All data will be documented and analyzed using SPSS software (SPSS Inc., Chicago, IL, USA), with the exception of the repeated-measures analysis, for which SAS software (SAS Institute, Cary, NC, USA) with the procedure PROC MIXED. No interim analysis will be performed.

## Discussion

Treatment of the axilla in patients with breast cancer has been a subject of recent debate. More than 50% of patients with a positive SLN appear to have no further LN involvement [20,21]. Based on these findings, the therapeutic role of cALND for these patients seems limited [22].

In the late 1990s, the American College of Surgeons Oncology Group (ACOSOG) Z0011 trial started in the USA. That trial randomized patients with breast cancer who had one to three positive SLNs to either cALND or a ‘wait and see’ policy. The results were recently published and no differences in overall survival (OS) or disease-free survival were seen after a median follow-up of 6.3 years [23]. Although the results do question the therapeutic benefit of ALND in SLNB-positive patients, abandoning ALND is not yet regarded as a standard of care. Moreover, considering the selected group of patients in the ACOSOG Z0011 trial, of which the majority received adjuvant chemotherapy and locoregional radiotherapy of the breast with inclusion of the lower regions of the axilla, the safety of omitting ALND in patients not fulfilling these criteria remains unclear.

A recently published study by Avril *et al*. [24] failed to show benefit in event-free survival and OS for post-menopausal patients with early breast cancer. This study randomized clinically node-negative post-menopausal patients with breast cancer to an ALND group or a no-ALND group. Adjuvant systemic therapy was based on the primary tumor characteristics.

In other studies, published several years ago, no difference was found in locoregional disease control, metastasis-free survival, and OS between patients treated with ALND and those treated with radiotherapy of the axilla [25]. However, as was the case with standard ALND, axillary radiotherapy appeared to be associated with an increased incidence of lymphedema [26,27].

The recently published Dutch guideline for breast-cancer treatment [28] gives several therapeutic options for the treatment of the axilla. It says that, in case of micrometastasis and macrometastasis with a maximum of two positive SLNs in patients receiving a breast-sparing therapy in combination with adjuvant systemic therapy, omission of the cALND can be considered. This may affect the accrual of the trial. However, there will be many patients left for whom pALND and cALND will remain the first-choice treatment; for example, in patients undergoing a mastectomy or in patients with a high risk of axillary involvement. For these patients, a limited and more tailored ALND based on the ARM technique might be a valuable tool to reduce the morbidity associated with the current ALND technique.

This multicenter randomized trial will provide evidence on whether or not upper-limb axillary LN preservation by means of ARM will decrease the side-effects of ALND.

## Trials status

This trial was designed in 2011 and 2012. The protocol passed through multiple amendments. Final approval from the medical ethics committee (METC) of Maxima Medisch Centrum, The Netherlands, was obtained on 21 September 2012 (METC number 1226: Centrale Commissie Mensgebonden Onderzoek (CCMO) number NL3920201512). This study will be carried out in compliance with the Helsinki Declaration. The trial is registered in the Dutch trial registration (TC 3698).

### Ethics

Patient recruitment will start in April 2013 in seven breast-cancer centers in the Netherlands. The study will finish enrolment as soon as 280 patients are included, and is expected to finish by April 2016. Collection and analysis of the results will be performed in the following 2 years.

## Abbreviations

ALND: Axillary lymph node dissection; ARM: Axillary reverse mapping; cALND: Complementary axillary lymph node dissection; CRF: Case report form; METC: Medical ethics review committee; pALND: Primary axillary lymph node dissection; PQ: Patient questionnaire; SLNB: Sentinel lymph node biopsy; WHOQOL: World Health Organization Quality of Life.

## Competing interests

This trial is funded by Pink Ribbon. The sponsor has no influence on the design of the protocol, patient recruitment, or data generation, and their participation will not affect the analysis of the results or writing of the manuscript(s). The authors have no financial of non-financial competing interest to declare.

## Authors’ contributions

EK participated in the design of the trial and data development, and drafted the manuscript. PG participated in the design of the trial, performed the database development, and participated in writing the manuscript. MD participated in the database development and critically revised the manuscript, and will be the leading researcher during the trial. AV participated in the design of the trial, performed the statistical analysis plan, and critically revised the manuscript. EL designed the trial and critically revised the manuscript. All authors read and approved the final manuscript.
